# Kalkitoxin Inhibits Angiogenesis, Disrupts Cellular Hypoxic Signaling, and Blocks Mitochondrial Electron Transport in Tumor Cells

**DOI:** 10.3390/md13031552

**Published:** 2015-03-20

**Authors:** J. Brian Morgan, Yang Liu, Veena Coothankandaswamy, Fakhri Mahdi, Mika B. Jekabsons, William H. Gerwick, Frederick A. Valeriote, Yu-Dong Zhou, Dale G. Nagle

**Affiliations:** 1 Department of BioMolecular Sciences and Research Institute of Pharmaceutical Sciences, School of Pharmacy, University of Mississippi, University, MS 38677, USA; E-Mails: jbmorga1@go.olemiss.edu (J.B.M.); 2007yl@gmail.com (Y.L.); ckveena@gmail.com (V.C.); fmahdi@umc.edu (F.M.); 2 Department of Biology, University of Mississippi, University, MS 38677, USA; E-Mail: jekabson@olemiss.edu; 3 Center for Marine Biotechnology and Biomedicine, Scripps Institution of Oceanography and Skaggs School of Pharmacy and Pharmaceutical Sciences, University of California San Diego, La Jolla, CA 920933, USA; E-Mail: wgerwick@ucsd.edu; 4 Department of Internal Medicine, Division of Hematology and Oncology, Henry Ford Hospital, Detroit, MI 48202, USA; E-Mail: fvaleri1@hfhs.org

**Keywords:** kalkitoxin, breast cancer, *Moorea producens*, mitochondria toxin, VEGF, angiogenesis inhibitor, hypoxia-inducible factor-1, HIF-1, *Lyngbya majuscula*

## Abstract

The biologically active lipopeptide kalkitoxin was previously isolated from the marine cyanobacterium *Moorea producens* (*Lyngbya majuscula*). Kalkitoxin exhibited *N*-methyl-d-aspartate (NMDA)-mediated neurotoxicity and acted as an inhibitory ligand for voltage-sensitive sodium channels in cultured rat cerebellar granule neurons. Subsequent studies revealed that kalkitoxin generated a delayed form of colon tumor cell cytotoxicity in 7-day clonogenic cell survival assays. Cell line- and exposure time-dependent cytostatic/cytotoxic effects were previously observed with mitochondria-targeted inhibitors of hypoxia-inducible factor-1 (HIF-1). The transcription factor HIF-1 functions as a key regulator of oxygen homeostasis. Therefore, we investigated the ability of kalkitoxin to inhibit hypoxic signaling in human tumor cell lines. Kalkitoxin potently and selectively inhibited hypoxia-induced activation of HIF-1 in T47D breast tumor cells (IC_50_ 5.6 nM). Mechanistic studies revealed that kalkitoxin inhibits HIF-1 activation by suppressing mitochondrial oxygen consumption at electron transport chain (ETC) complex I (NADH-ubiquinone oxidoreductase). Further studies indicate that kalkitoxin targets tumor angiogenesis by blocking the induction of angiogenic factors (*i.e.*, VEGF) in tumor cells.

## 1. Introduction

When environmental factors are conducive, the benthic marine cyanobacterium *Moorea producens* sp. nov. (Oscillatoriaceae), previously classified as *Lyngbya majuscula* Gomont, typically forms localized mini-blooms that may cover sections of a coral reef [1,2,3,4]. Marine cyanobacteria produce a wide variety of structurally novel secondary metabolites [5,6]. Most of the more than 300 natural products isolated from *M. producens* can be classified into one of two general biosynthetic classes: (A) non-ribosomally produced linear and cyclic peptides, and cyclic depsipeptides, and (B) lipopeptides of mixed biogenetic origin that combine polyketide or fatty acid derived precursors with peptide-derived structural components [6].

**Figure 1:**
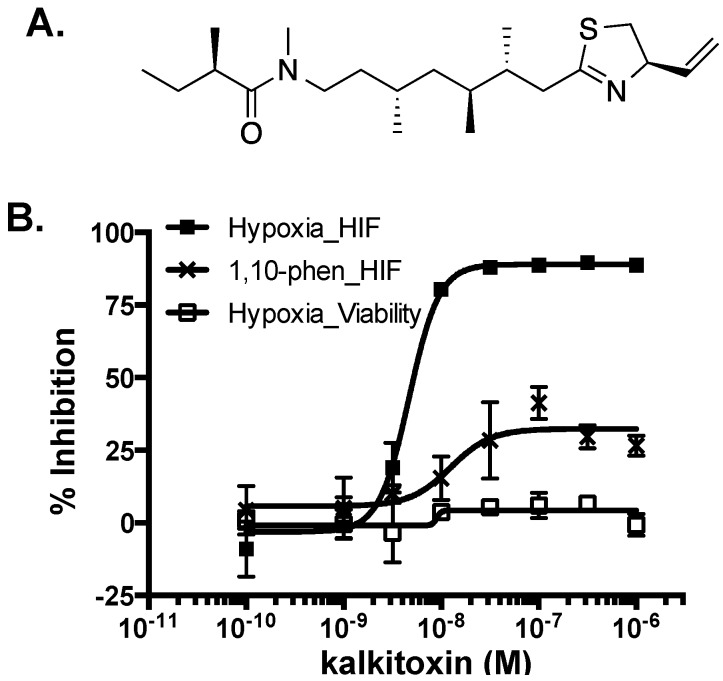
(**A**) Structure of kalkitoxin; (**B**) Kalkitoxin is a potent inhibitor of hypoxia-induced HIF-1 activation. Exponentially grown T47D cells transfected with the pHRE3-TK-luc construct for HIF-1 activity were plated into 96-well plates. Kalkitoxin was added at the specified concentrations and the cells exposed to hypoxia (1% O_2_) or 1,10-phenanthroline (10 µM) for 16 h, respectively. Cells were lysed, luciferase activity determined, and the data presented as “% Inhibition” of the induced control. Data shown are average ± standard deviation (*n* = 3). For the viability study, T47D cells plated into 96-well plates were exposed to kalkitoxin and hypoxia as that described for the reporter assay. Cell viability was determined by the SRB method. Data presentation is the same as that described for the reporter assay.

The lipopeptide kalkitoxin (Figure 1A) was first isolated from a Curaçao collection of *M. producens* [7,8] and found to be ichthyotoxic (*Carassius auratus*, LC_50_ 700 nM) and brine shrimp toxic (*Artemia salina*, LC_50_ 170 nM), inhibit cell division (fertilized sea urchin embryo assay, IC_50_ ~50 nM), suppress inflammation, and potently block voltage sensitive-sodium channels in murine neuro-2a cells (EC_50_ 1 nM). Synthetic kalkitoxin analogues were also highly toxic to *A. salina* [9]. Kalkitoxin displayed exposure time-dependent potent neurotoxicity towards primary rat cerebellar granular neurons (CGNs) (LC_50_ 3.86 nM) [10]. Mechanistic studies examined the interaction of kalkitoxin with the tetrodotoxin- and voltage-sensitive sodium channel (TTX-VSSC) in CGN cells [11]. Total synthesis and biological evaluation of (+)-kalkitoxin, the naturally occurring form, revealed that kalkitoxin displayed solid tumor-selective cytotoxicity when evaluated in extended duration clonogenic assays (colorectal carcinoma HCT-116 cells: 10% survival at 0.002 µg/mL with 168 h exposure; inactive at 10 µg/mL with 24 h exposure) [12]. However, the molecular mechanism(s) responsible for the potent tumor cell-selective cytotoxicity was unclear.

## 2. Results and Discussion

### 2.1. HIF-1 Inhibitory Activity

The transcription factor hypoxia-inducible factor-1 (HIF-1) regulates oxygen homeostasis by activating the expression of genes that increase oxygen availability and those that decrease oxygen consumption, thus mediating cellular adaptation to hypoxia [13]. Preclinical and clinical studies have established that HIF-1 dysregulation directly impacts cancer etiology and progression, while HIF-1 inhibition suppresses tumor growth and enhances the efficacy of both radiation and chemotherapy [14,15,16]. As part of our ongoing campaign to identify natural product-based inhibitors of HIF-1 activation, a human breast tumor T47D cell-based HIF-1 reporter assay was used to evaluate ~300 purified marine natural products and 15,000 marine invertebrate and algae extracts from the U.S. National Cancer Institute’s (NCI’s) Open Repository [17,18,19,20]. Kalkitoxin (1 µM) completely inhibited HIF-1 activation in the primary screening. Concentration-response studies were performed in T47D cells to determine the effects of kalkitoxin on HIF-1 activation. At low nanomolar concentrations, kalkitoxin selectively blocked hypoxia-induced HIF-1 activation (IC_50_ 5.7 nM, 95% CI: 4.6 to 7.1 nM, Figure 1), relative to its effect on chemical hypoxia (1,10-phenanthroline; 10 µM)-activated HIF-1 (IC_50_ > 1 µM, Figure 1). Parallel viability assay results indicated that kalkitoxin inhibited hypoxic HIF-1 activation without pronounced cytotoxicity, even up to micromolar levels at the 16 h time point (Figure 1).

### 2.2. Suppression of HIF-1 Target Gene Expression

**Figure 2:**
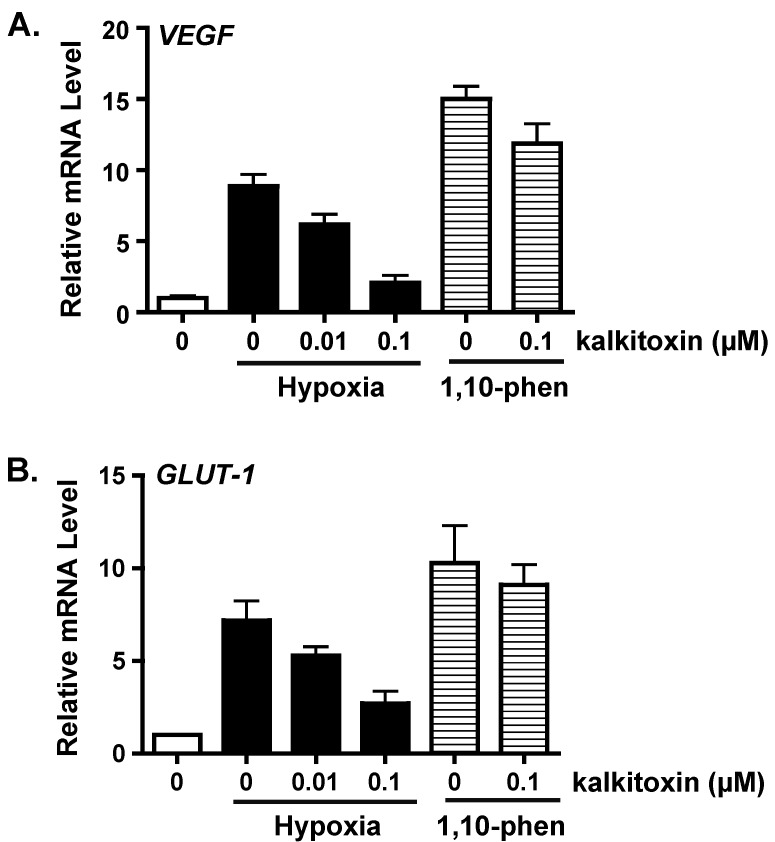
Kalkitoxin blocks hypoxic induction of HIF-1 target genes *VEGF* and *GLUT-1* at the mRNA level. Kalkitoxin was added to exponentially grown T47D cells at the specified concentrations and the incubation continued for another 16 h under hypoxia (1% O_2_) or in the presence of 1,10-phenanthroline (1,10-phen, 10 µM), respectively. The cells were lysed and total RNA extracted. The levels of *VEGF* (**A**) and *GLUT-1* mRNAs (**B**) were determined by quantitative real time RT-PCR, normalized to that of an internal control 18S rRNA, and presented as relative values to an untreated control determined by the ΔΔC_T_ method (mean ± standard deviation, *n* = 3).

As a key regulator of oxygen homeostasis, HIF-1 controls the expression of over one hundred genes that modulate critical aspects of cellular physiology [13]. The effects of kalkitoxin on the induction of HIF-1 target genes *VEGF* (vascular endothelial growth factor) and *GLUT-1* (glucose transporter-1) were examined by real time RT-PCR. Hypoxic exposure of T47D cells (1% O_2_, 16 h) increased the expression of *VEGF* (Figure 2A) and *GLUT-1* (Figure 2B) at the mRNA level. Kalkitoxin (0.01 and 0.1 µM) inhibited the hypoxic induction of *VEGF* or *GLUT-1* mRNA expression in a concentration-dependent manner (Figure 2). As observed in the T47D cell-based HIF-1 reporter assays (Figure 1B), the inhibitory effects exerted by kalkitoxin were significantly greater for HIF-1 target genes that were induced by hypoxia, compared to those induced by 1,10-phenanthroline (10 µM) (Figure 2).

### 2.3. Inhibition of Hypoxia-Induced Angiogenesis

**Figure 3:**
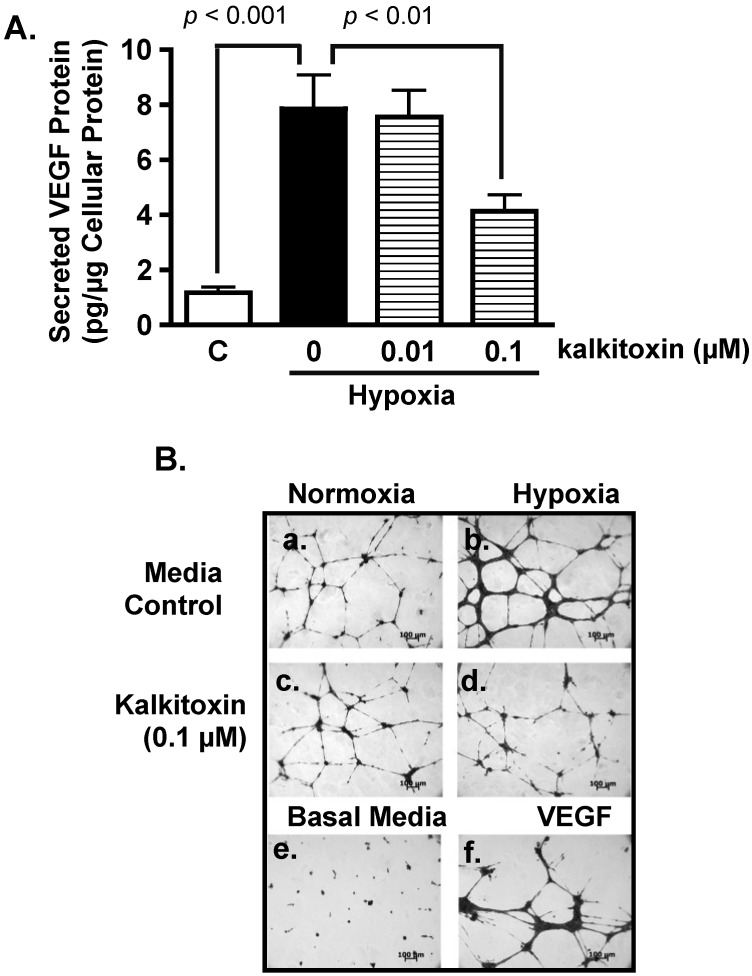

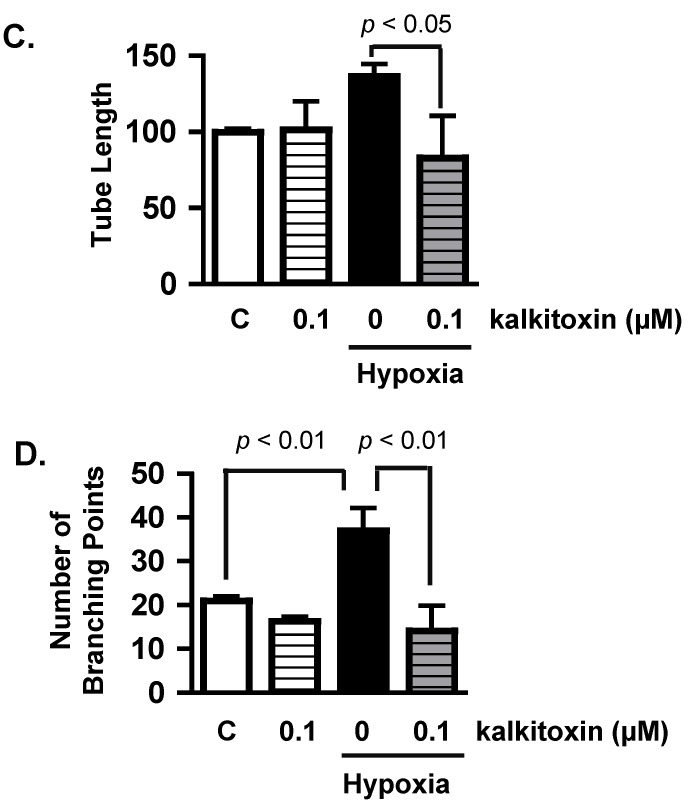
Kalkitoxin inhibits hypoxia-stimulated tumor angiogenesis by blocking the induction of angiogenic factor VEGF. (**A**) T47D cells were exposed to hypoxic conditions (1% O_2_, 16 h) in the presence and absence of kalkitoxin at the specified concentrations. The levels of secreted VEGF protein in the conditioned media samples were determined by ELISA and normalized to the amount of total cellular proteins (average + standard deviation, *n* = 3). “C”—media control. The *p* values of statistically significant differences when compared to the media controls are shown; (**B**) Representative HUVEC tube formation assay results. T47D cell-conditioned media samples were prepared as described in (A). Both negative (Basal Media, **e**) and positive (VEGF, **f**) controls are included at the bottom. A 100 µm scale bar is included in each panel; (**C**) Average + standard deviation of tube length, quantified from three randomly selected fields for each specified condition. The *p*-values are shown for statistically significant differences; (**D**) Branching points, data determined and presented as described in (C).

Hypoxic regions are commonly found throughout solid tumors. The extent of tumor hypoxia correlates with advanced disease stages and treatment resistance among cancer patients [21,22,23]. To survive in a low O_2_ environment, hypoxic tumor cells stimulate tumor angiogenesis through the HIF-1-dependent induction of the potent angiogenic factor, VEGF [24]. Hypoxic exposure (1% O_2_, 16 h) of T47D cells significantly increased the production of secreted VEGF protein, relative to the untreated control (Figure 3A). Kalkitoxin (0.1 µM) suppressed the level of hypoxia-induced secreted VEGF protein by ~50% (Figure 3A). The potential anti-angiogenic activity of kalkitoxin was assessed using a human umbilical vein endothelial cell (HUVEC)-based tube formation assay, a widely employed *in vitro* model for angiogenesis. Under normal culture conditions, HUVEC cells appear scattered (Basal Media, Figure 3B, panel e). When exposed to angiogenic factors, such as recombinant human VEGF protein, HUVEC cells are stimulated to form interconnected tube-like structures (tube formation, VEGF, Figure 3B, panel f) [25]. Tube formation was induced by normoxic T47D cell-conditioned media (Control/Normoxia, Figure 3B, panel a). The angiogenic activity of the T47D cell-conditioned media sample was significantly enhanced by hypoxic exposure (1% O_2_, 16 h) (Control/Hypoxia, Figure 3B, panel b), which increases angiogenic factor (*i.e.*, VEGF) production (Figure 3A). Kalkitoxin (0.1 µM) suppressed the angiogenic activity of the hypoxic T47D cell-conditioned media at a concentration that also inhibited hypoxia-induced HIF-1 activation and VEGF induction (Kalkitoxin/Hypoxia, Figure 3B, panel d). Kalkitoxin did not prevent normoxic T47D cell-conditioned media from inducing angiogenesis (Kalkitoxin/Normoxia, Figure 3B, panel c). Thus, kalkitoxin appears to inhibit tumor angiogenesis by blocking the induction and/or expression of angiogenic factors, not by directly suppressing the tube formation process. These observations are corroborated by quantitative microscopic results (Figure 3C, tube length; Figure 3D, number of branching points).

### 2.4. Mechanism of Action Studies

#### 2.4.1. HIF-1α Expression

**Figure 4:**
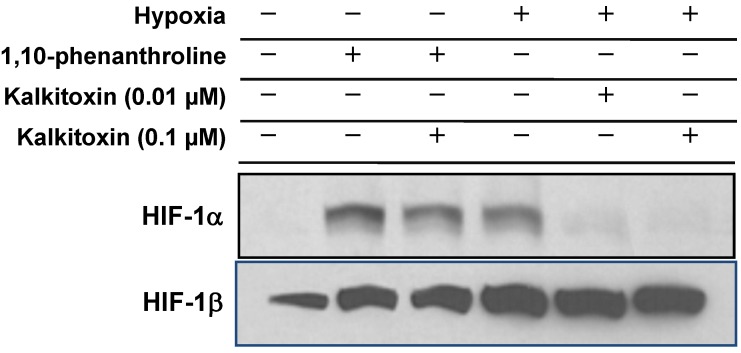
Kalkitoxin selectively inhibited the induction of HIF-1α protein by hypoxia. T47D cells were exposed to kalkitoxin at the specified concentrations, under hypoxic conditions (1% O_2_, 4 h) or in the presence of 1,10-phenanthroline (10 µM, 4 h). Nuclear extract samples were prepared from both untreated control and treated cells, and the levels of HIF-1α and HIF-1β proteins determined by Western blot.

To discern the mechanism(s) responsible for the inhibition exerted by kalkitoxin on HIF-signaling, its effect on nuclear HIF-1α protein induction was assessed in T47D cells. In general, HIF-1 activity is determined by the concurrent induction and activation of the oxygen-regulated HIF-1α subunit [26]. The nuclear extract from control cells under normoxic conditions did not contain HIF-1α protein (Figure 4). Hypoxic conditions (1% O_2_, 4 h) induced nuclear HIF-1α protein accumulation (Figure 4). Kalkitoxin (0.01 and 0.1 µM) blocked the hypoxic induction of HIF-1α protein, without affecting constitutively expressed HIF-1β subunit levels in the nuclear extract samples from hypoxia-exposed cells (Figure 4). In contrast, kalkitoxin did not block the induction of HIF-1α protein by 1,10-phenanthroline under normoxic conditions (10 µM 1,10-phen, 4 h, Figure 4).

#### 2.4.2. Mitochondrial Respiration Studies

**Figure 5:**
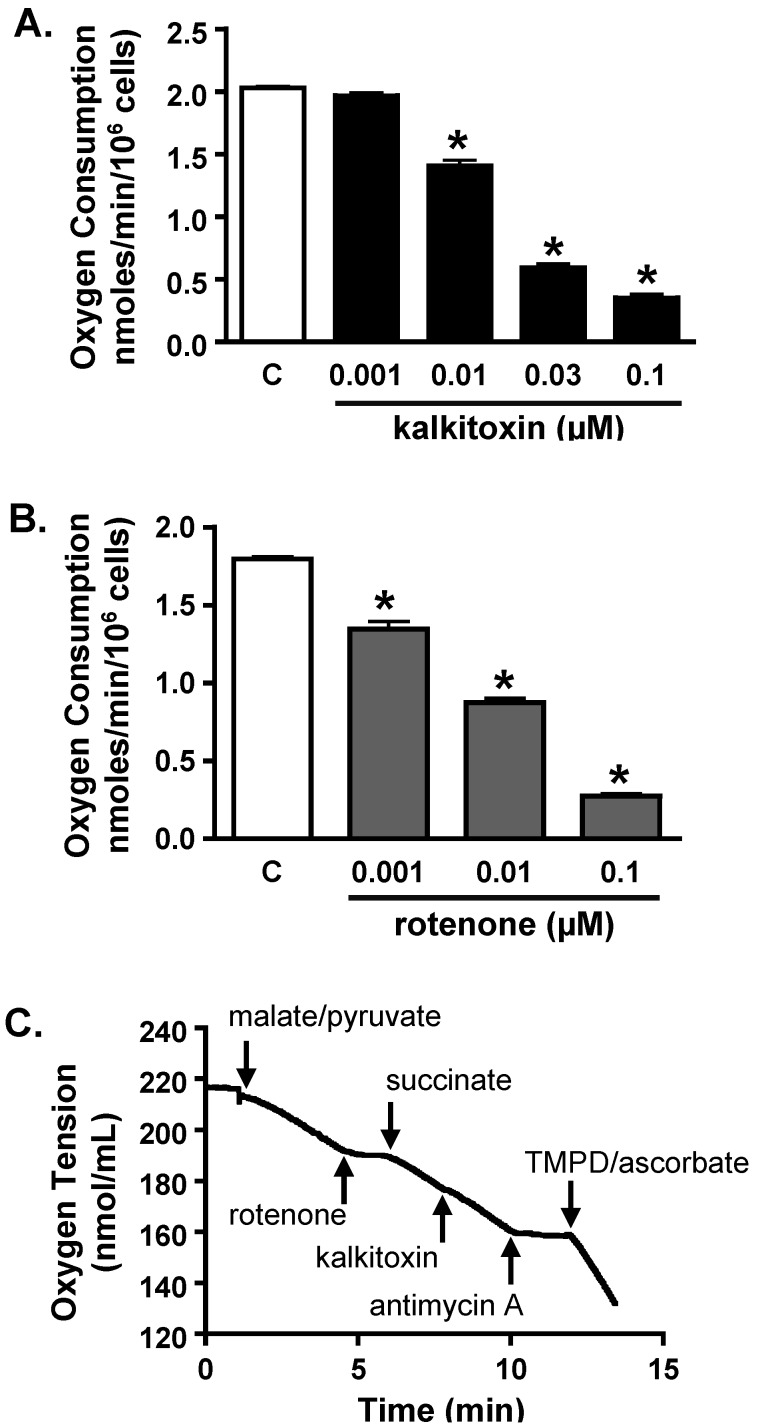

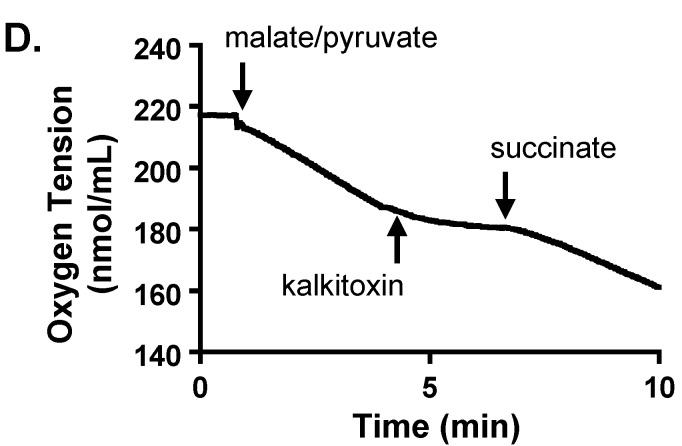
Kalkitoxin inhibits mitochondrial respiration by targeting ETC complex I. (**A**) Kalkitoxin was added to T47D cells at the specified concentrations and the rates of oxygen consumption determined using a Clark-type oxygen electrode. Data shown are average + standard deviation from three independent experiments. An “∗” indicates statistically significant difference when compared to the untreated control (“C”); (**B**) Concentration-response of rotenone on T47D cell respiration. Data presentation in (B) is the same as described in (A); (**C**,**D**) Mitochondrial substrates and inhibitors were added to digitonin-permeabilized T47D cells in the specified sequential order and the rates of oxygen consumption measured.

Mitochondrial electron transport chain (ETC) inhibitors selectively suppress hypoxia-induced HIF-1 activation [27,28,29,30]. The effect of kalkitoxin on mitochondrial function was examined in a T47D cell-based respiration assay [17,25]. The level of respiration correlates with the rate of oxygen consumption by the cell. Kalkitoxin inhibited oxygen consumption within the same range of concentrations that inhibited hypoxia-induced HIF-1 activation (Figure 5A). The potency for kalkitoxin to suppress cellular respiration is comparable to that observed for the ETC complex I inhibitor rotenone (Figure 5B). Mechanistic studies were performed to discern the specific site that kalkitoxin targets within the mitochondrial ETC. Kalkitoxin was first tested to see if it acts as an inhibitor of ETC complex II, III, or IV (Figure 5C). Digitonin-permeabilized T47D cells were treated with a mixture of malate and pyruvate to start respiration by initiating NADH production, thereby providing a source of electrons for complex I (NADH-ubiquinone oxidoreductase). The electrons then pass through a series of ETC electron carriers to oxygen that acts as the end electron acceptor. As anticipated, the standard complex I inhibitor rotenone (1 µM) inhibited oxygen consumption (Figure 5C). The ETC complex II (succinate-ubiquinone oxidoreductase) substrate succinate overcame rotenone-stalled respiration by shuttling electrons to complex III. Because kalkitoxin (30 nM) did not inhibit respiration in the presence of succinate, kalkitoxin did not appear to inhibit complex II, III, or IV (Figure 5C). In contrast, antimycin A (1 µM) [an inhibitor of complex III (ubiquinol-cytochrome c oxidoreductase)] suppressed respiration in the presence of succinate. A mixture of ascorbate and TMPD (*N*,*N*,*N*′,*N*′-tetramethyl-*p*-phenylenediamine) that serves as an electron source for cytochrome c and hence for complex IV (cytochrome c oxidase) resumed respiration that was blocked by antimycin A at complex III. These control experiments indicate that each of the ETC complexes II, III, or IV remain functional and are not affected by kalkitoxin treatment. To confirm that kalkitoxin inhibits mitochondrial respiration by selectively targeting complex I, kalkitoxin (30 nM) was added to permeabilized T47D cells following respiration initiation by a malate/pyruvate mixture. Kalkitoxin decreased respiration and succinate overcame this inhibition by shuttling electrons directly to complex III, thus circumventing the inhibitory effect of kalkitoxin on complex I (Figure 5D). Taken together, these results indicate that kalkitoxin potently suppresses mitochondrial respiration in tumor cells by selectively inhibiting ETC complex I.

### 2.5. Tumor Cell Proliferation/Viability

**Figure 6:**
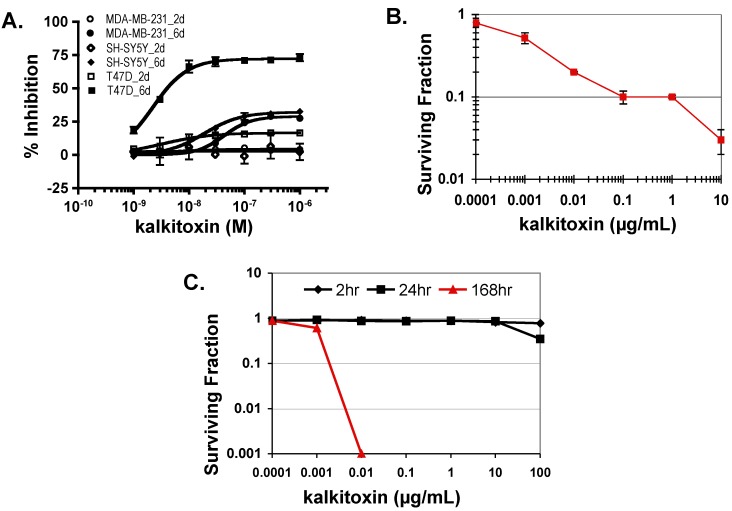
Kalkitoxin suppresses tumor cell proliferation/viability in a cell line- and time-dependent manner. (**A**) Exponentially grown T47D, MDA-MB-231, and SH-SY5Y cells were exposed to kalkitoxin at the specified concentrations for 48 h (2 days) and 144 h (6 days), respectively. Cell viability was determined by the SRB method and presented as “% Inhibition” of the untreated control (average ± standard deviation, *n* = 3); (**B**) HCT116 cells were exposed to kalkitoxin at the specified concentrations for 5 days and the number of surviving cells determined by trypan blue exclusion. Surviving fraction data are presented as the average ± standard deviation (*n* = 3); (**C**) Following kalkitoxin treatment for 2, 24, and 168 h at the specified concentrations, HCT116 cells were detached and plated at low density. Seven days later, the number of colonies was counted and the surviving fraction data presented (average ± standard deviation, *n* = 3).

In general, standard cytostatic/cytotoxic assays of 48 h duration are performed to evaluate the anticancer potential of active leads [31]. Our studies and those of McLaughlin and coworkers’ indicate that an extended exposure time (e.g., six days) is required to observe the full impact of mitochondrial ETC inhibitors on tumor cell proliferation and/or viability [28,32]. To determine the effects of kalkitoxin on tumor cell proliferation/viability, concentration-response studies were performed following both standard and extended exposure schedule (48 h and 144 h, respectively). Enhanced inhibition was observed with all three-cell lines (human breast cancer T47D and MDA-MB-231, and neuroblastoma SH-SY5Y) in the extended exposure study (Figure 6A). The most pronounced increase was observed in T47D cells (Figure 6A). Additionally, exponentially grown HCT116 cells were exposed to kalkitoxin at the specified concentrations for five days and the surviving cells monitored using the trypan blue excluding method. Kalkitoxin decreased HCT116 survival with an IC_50_ value of 1 ng/mL (or 2.7 nM, Figure 6B). The impact of kalkitoxin on tumor cell survival was further examined in a clonogenic assay. HCT116 cells were exposed to kalkitoxin for the specified amount of time (2 h, 24 h, and 168 h, respectively), and the ability of treated cells to form colonies determined (Figure 6C). While little if any cell killing occurred for either a 2 or 24 h exposure for concentrations up to 100 µg/mL (270 µM), an extended exposure (168 h) to kalkitoxin was required to significantly suppress tumor cell colony formation, similar to that observed in Figure 6B.

### 2.6. Neurotoxicity

**Figure 7:**
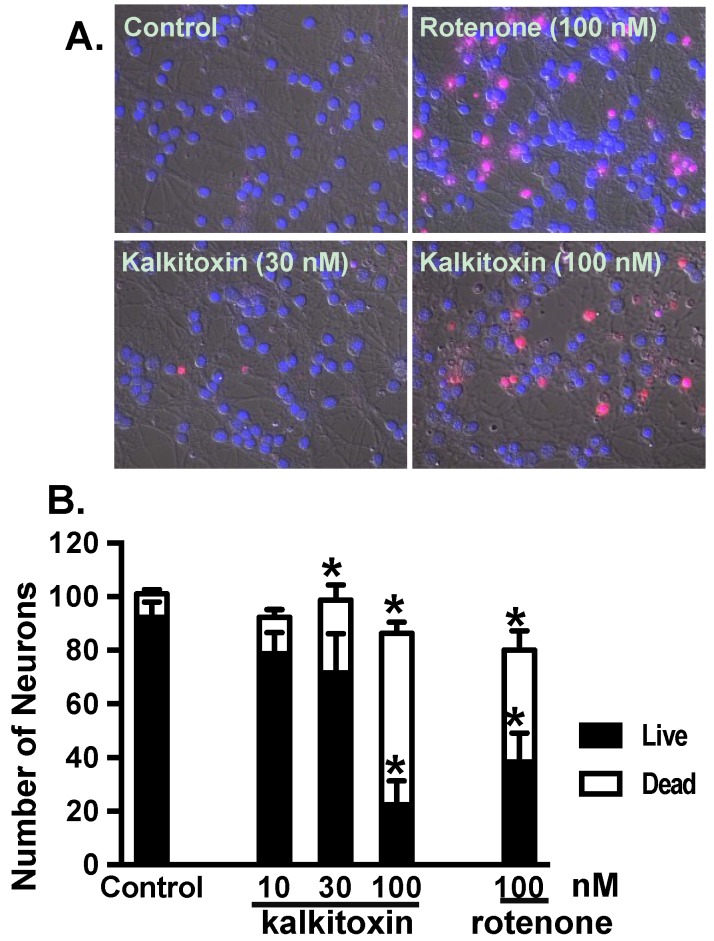
alkitoxin induces neurotoxicity *in vitro*. (**A**) Representative images of PI and Hoechst-33342 stained rat CGNs exposed to media (control) and kalkitoxin (30 and 100 nM, respectively) for 24 h; (**B**) The extent of cell death was quantified by counting live and dead (PI positive) neurons in four randomly selected fields for each specified condition. Data shown are mean + standard error (*n* = 8), pooled from two independent experiments. An “∗” indicates statistically significant difference when compared to the untreated control.

Exposure to certain mitochondrial inhibitors is associated with neurotoxicity [33,34]. Kalkitoxin was evaluated for potential neurotoxicity using primary rat cerebellar granule neurons (CGNs) as an in vitro model. Following compound treatment (24 h), CGNs were stained with propidium iodide (PI) for dead cells and Hoechst-33342 for all cells. Because PI does not penetrate intact cell membranes, it stains both late stage apoptotic and necrotic cells (Figure 7A). The cells were counted and grouped, based on morphological characteristics (live *versus* dead, Figure 7B). Relatively high kalkitoxin concentrations (*i.e.*, 100 nM, 24 h) killed most cells, but intermediate concentrations (*i.e.*, 30 nM) induced only moderate cytotoxicity (Figure 7B). The neurotoxicity incurred by kalkitoxin is comparable to that observed with the positive control, mitochondrial ETC complex I inhibitor, rotenone (Figure 7B). Kalkitoxin was reported to incur CGN toxicity with a LC_50_ value of 3.86 nM [9]. Treatment condition-associated stress (e.g., 22 °C *versus* 37 °C, Locke’s buffer *versus* culture media, *etc.*) may have enhanced the neurotoxic effect of kalkitoxin in the previous studies.

## 3. Experimental Section

### 3.1. Tumor Cell Culture, Cell-Based Reporter and Viability Assays

The T47D, MDA-MB-231, SH-SY5Y, and HCT116 cells were from ATCC. Cells were maintained in DMEM/F12 media with l-glutamine (Mediatech, Manassas, VA, USA), supplemented with 10% (v/v) fetal bovine serum (FBS, Hyclone, Logan, UT, USA), 50 units/mL penicillin and 50 µg/mL streptomycin (Gibco, Grand Island, NY, USA). To monitor HIF-1 activity, a T47D cell-based luciferase assay employing the pHRE3-TK-Luc reporter was performed as previously described [17]. Except for the HCT116 studies, the sulfarhodamine B method was used to determine cell viability [25]. For the extended duration six-day exposure study, the conditioned media were replaced after three days by fresh culture medium that contained test compound. The HCT116 cell-based IC_50_ and clonogenic studies were performed as previously described [35]. Test compounds were prepared as stock solutions in DMSO or isopropanol as appropriate and stored at −20 °C. In general, the final solvent concentration was less than 0.5% (v/v).

### 3.2. RNA Extraction and Quantitative Real Time RT-PCR

Experimental design, detailed procedures, and data analysis were as previously reported [25].

### 3.3. ELISA Assay for Human VEGF Protein

T47D cell plating, compound treatments, and ELISA assay for secreted VEGF proteins were as previously reported [17]. Proteins concentrations in the cellular lysate were determined using a micro BCA assay (PIERCE), and the secreted VEGF protein levels were normalized to those of cellular proteins.

### 3.4. HUVEC-Based Tube Formation Assay

The maintenance of HUVEC cells (Lonza, Walkersville, MD, USA), T47D cell-conditioned media (CM) sample collection, and the HUVEC-based *in vitro* tube formation assays were performed and data quantified as described previously [25].

### 3.5. Nuclear Extract Preparation and Western Blot Analysis

Preparation of nuclear extract samples from both treated and control T47D cells, and determination of HIF-1α and HIF-1β proteins by Western blot were described previously [25].

### 3.6. Mitochondria Respiration Assay

The oxygen consumption rates of T47D cells were monitored using an Oxytherm Clarke-type electrode System (Hansatech, Norfolk, UK). The effects of purified compounds on cellular respiration were determined using a non-permeabilized cell-based respiration assay [25]. Mechanistic studies were conducted in digitonin-treated cells with permeabilized plasma membrane to manipulate mitochondrial substrates and inhibitors. Detailed experimental procedures and reagents were as previously described [25].

### 3.7. Cerebellar Granule Neuron Preparation and Neurotoxicity Assay

Experiments that involved the use of rat-derived materials were approved by the Institutional Animal Care and Use Committee, University of Mississippi (File Number 06-009, approved on 25 October 2005), and were handled in strict accordance with good animal practice as defined by the NIH guidelines. Detailed experimental procedures and reagents were as previously described [28].

### 3.8. Statistical Analysis

Data were compared using one-way ANOVA followed by Bonfferoni post hoc analyses (GraphPad Prism 4). Differences were considered statistically significant when *p* < 0.05.

## 4. Conclusions

**Figure 8:**
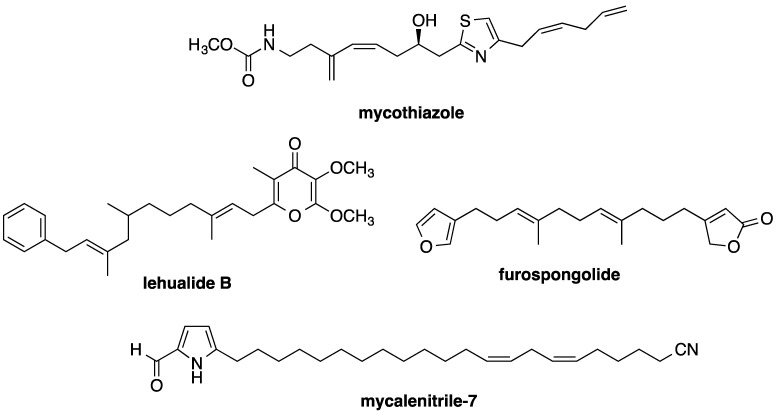
Examples of marine invertebrate metabolites that have recently been found to inhibit mitochondrial electron transport chain complex I and disrupt HIF-mediated hypoxic signaling in tumor cells.

Sodium channel and mitochondria-associated neurotoxicity may limit the therapeutic potential of kalkitoxin as an antitumor chemotherapeutic agent. Mechanistically, kalkitoxin and other recently reported marine natural product HIF-1 inhibitors (Figure 8) suppress the multi-enzyme mitochondrial NADH-ubiquinone oxidoreductase system (electron transport chain complex I), thus disrupting mitochondria-mediated hypoxic signaling. Examples of mitochondria-targeted HIF-1 inhibitors include an assortment of sponge metabolites (e.g., mycothiazole [28], lehualide B [36], furospongolide [37], and the mycalinitriles [38]), and structurally dissimilar algal natural products [39,40]. Kalkitoxin production by the marine cyanobacterium, *Moorea producens*, is physiologically and ecologically intriguing. The propensity of kalkitoxin to potently and selectively block both voltage-sensitive sodium channels and complex I of the mitochondrial electron transport chain appears to be unnecessarily redundant means of chemical defense. The production of potent sodium channel-targeted toxins would seem to impart a more than adequate defense against herbivorous grazers. However, recent studies indicate that a number of marine invertebrates and fish species have evolved altered sodium channels with reduced sensitivity to TTX-VSSC-targeted toxins (reviewed in [41]). This is not particularly surprising, considering the potential for regular exposure of marine filter feeders and grazers to algae- and bacteria-derived sodium channel inhibitors (e.g., saxitoxin and tetrodotoxin), and adaptive pressure for the presumably defensive physiological accumulation of these neurotoxins in the tissues of invertebrate [e.g., blue-ringed octopus (*Hapalochlaena* spp.)] and vertebrate species [e.g., pufferfish (*Diodon* spp.)]. Thus, by simultaneously acting as both a voltage-gated Na^+^ channel blocker and a potent rotenone-like mitochondrial disruptor, kalkitoxin may be able to provide an additional level of grazing defense against a broad range of herbivore species.
